# FusionEP: Adaptive Cross-Modal Deep Fusion Network for Essential Protein Identification via Multi-Omics Integration and Contrastive Learning

**DOI:** 10.3390/e28101117

**Published:** 2026-10-13

**Authors:** Chuanyan Wu, Bentao Lin, Qingju Zhang, Jialin Zhang, Rui Gao, Zhi-Ping Liu

**Affiliations:** 1 School of Intelligent Engineering, Shandong Management University, No. 3500 Dingxiang Road, Jinan 250357, China; chuanyan_wu@sdmu.edu.cn (C.W.); linbentao@sdmu.edu.cn (B.L.); 2 School of Intelligent Equipment, Shandong University of Science and Technology, Taian 271019, China; 3 School of Control Science and Engineering, Shandong University, No. 17923 Jingshi Road, Jinan 250061, China; jl_zhangsdu@163.com (J.Z.); gaorui@sdu.edu.cn (R.G.)

**Keywords:** essential protein prediction, contrastive learning, data fusion, multi-omics integration

## Abstract

Identifying essential proteins lays a foundation for dissecting disease mechanisms and mining therapeutic targets. Existing algorithms mostly adopt unimodal inputs and struggle to capture multi-level biological interactions from genomics, subcellular localization and proteomics. We present FusionEP, which is an adaptive cross-modal deep fusion network built upon contrastive learning to predict essential proteins. FusionEP integrates gene expression profiles, subcellular localization features, and protein interaction map structures. For gene expression information, we design a six-layer Transformer with 12 attention heads and learnable positional encodings for feature extraction. For subcellular features, a multi-scale convolutional neural network (CNN) with four kernel sizes and a channel spatial attention mechanism is designed for feature extraction. A four-layer Graph Attention Network (GAT) with dynamic attention, residual connections, and target node readout functionality is designed to extract feature from protein–protein interaction (PPI) networks. FusionEP employs an adaptive fusion gate for dynamic feature aggregation and supervised contrastive learning for cross-modal alignment. Furthermore, prototype-based classification with focal loss optimization is used to address class imbalance. We evaluated the model’s performance on the yeast biological dataset. The results showed that FusionEP achieved an AUC-ROC of 0.9523 ± 0.003 in five independent experiments. FusionEP provides an end-to-end solution for multimodal protein analysis. This work sets a new method for integrating heterogeneous biological data in precision medicine applications.

## 1. Introduction

Essential proteins play a pivotal role in sustaining cellular survival, regulating disease evolution, and supporting therapeutic treatment development, making their accurate identification a core research problem in systems biology and drug development [1,2]. In protein–protein interaction (PPI) networks, essential proteins commonly serve as critical hub nodes, possessing distinct topological structures and functional properties that differentiate them from non-essential protein molecules [3].

Traditional experimental approaches, including RNA interference (RNAi) screening and CRISPR-mediated gene knockout assays, can achieve reliable results in essential protein verification. Nevertheless, these experimental techniques suffer from inherent drawbacks such as lengthy experimental cycles, heavy manual workloads, high economic costs, and poor expandability, which greatly restrict their large-scale application in high-throughput protein analysis [2].

With the rapid advancement of high-throughput biological detection technologies, massive and heterogeneous biological datasets have been accumulated, covering PPI network structures, gene expression patterns, and protein subcellular localization profiles [4]. Mining and identifying essential proteins from these multi-source complementary data has become a key research direction in systems biology, driving scholars to develop a variety of computational prediction methods for essential protein recognition.

Initial computational research on essential protein prediction mainly depended on network topological features. Classic topological metrics, including degree centrality [3], betweenness centrality [5], closeness centrality [6], and eigenvector centrality [7], have been extensively adopted for protein essentiality evaluation, and graph-based analytical methods have also been proven effective in screening biologically significant proteins [8]. Even so, topology-dependent methods rely heavily on artificially constructed features, which limits their capability to capture the intricate nonlinear correlation relationships among proteins. To compensate for the deficiencies of traditional topological methods, machine learning algorithms have been gradually applied to essential protein prediction tasks. Supervised learning models are capable of autonomously extracting discriminative feature patterns from biological data. In our previous study, we constructed a prediction framework integrating ReliefF feature selection and random forest classification for essential protein identification [9]. In recent years, ensemble learning strategies have also been introduced to optimize prediction performance. For instance, Inzamam-Ul-Hossain and colleagues adopted genetic algorithm-based feature selection combined with multiple classifiers, including random forest, LightGBM and XGBoost to boost the accuracy of essential protein prediction [10]. However, the prediction performance of such machine learning methods is still constrained by manually designed feature systems. The emergence of deep learning techniques has greatly enhanced the modeling capacity for complex biological network data. Graph Neural Networks (GNNs), in particular, have become dominant analytical tools for PPI network mining [11]. As a representative deep learning model, DeepEP integrates node2vec feature embedding, multi-scale convolutional neural networks and network sampling strategies to realize efficient essential protein detection [12]. Despite the promising progress of existing deep learning-based methods, most models only focus on local neighbor feature aggregation of network nodes, failing to fully exploit global biological information.

The abundance of multi-source heterogeneous biological data has further promoted the development of multimodal learning paradigms in protein research. Compared with single-modal analytical methods, multimodal models that fuse complementary biological information can achieve more comprehensive and fine-grained protein feature representation [13]. In a recent study, Ye et al. proposed a novel prediction framework, which fuses time-series gene expression data, PPI network topology and subcellular localization information, and adopts evolutionary community detection and SVM-RFE feature selection strategies to complete essential protein prediction [14]. In our previous work, we employed GCN-GAT networks, BiLSTM, and deep neural networks to extract structural, temporal, and biological features from PPI networks, gene expression data, and subcellular localization information, respectively. Self-attention and cross-attention mechanisms were subsequently adopted to fuse multimodal representations before classification [15]. Although attention-based fusion strategies can dynamically adjust modality contributions, they often assume that multimodal representations are already aligned within a shared semantic space.

Recently, contrastive learning has attracted considerable attention in representation learning because of its ability to improve feature discriminability and semantic consistency across different views of the same sample [16]. Contrastive learning has achieved remarkable success in multimodal learning and various bioinformatics applications. Nevertheless, its application to essential protein prediction remains relatively limited. In particular, the alignment of heterogeneous biological modalities, including graph-structured, tabular, and spatial biological data, has not been sufficiently investigated.

Experimental techniques such as RNA interference (RNAi) screening and clustered regularly interspaced short palindromic repeats (CRISPR)-based gene-knockout assays can identify essential proteins with high accuracy, yet they are labor-intensive and costly. High-throughput multi-omics resources such as PPI networks, gene expression measurements and subcellular localization annotations create opportunities to build computational prediction pipelines for essential proteins. Benchmark annotations of essential proteins are typically compiled by merging records from several public repositories, namely the Saccharomyces Genome Database (SGD), Munich Information Center for Protein Sequences (MIPS), Database of Essential Genes (DEG), and Saccharomyces Genome Deletion Project (SGDP).

Despite considerable progress, several key challenges remain unresolved. First, full-batch graph-neural-network training on large-scale PPI networks suffers from high computational cost. Second, effective semantic alignment across heterogeneous multi-modal biological data remains difficult. Third, graph-based models frequently ignore global topological importance metrics. Fourth, severe class imbalance exists because essential proteins constitute less than 20% of all proteins [17].

To address these issues, we propose FusionEP, an adaptive cross-modal deep fusion network enhanced by subgraph sampling and supervised contrastive learning. Our model employs modality-specific encoders, uncertainty-aware adaptive feature fusion, cross-modal supervised contrastive alignment, and prototype-regularized classification with focal-loss optimization for end-to-end essential-protein prediction. We employ a modality-specific encoder and an adaptive fusion gate for dynamic feature aggregation. Simultaneously, supervised contrastive learning is used for cross-modal alignment. To address the class imbalance problem, we utilize prototype-based classification and focal loss. The main contributions are as follows. First, we propose a *k*-hop subgraph sampling strategy to capture global topological importance without processing the entire network. This strategy extracts the local neighborhood structure around the target protein. It also incorporates network centrality features including degree centrality, betweenness centrality, proximity centrality, and eigenvector centrality. Secondly, we designed a dedicated encoder for each biological modality. Gene expression data employed a Transformer-based architecture for feature extraction. Subcellular localization data utilized a multi-scale CNN with channel spatial attention for feature extraction. Graph structure protein data were extracted using a deep GATv2 algorithm with residual connections. Third, this work incorporates cross-modal contrastive learning to sustain discriminative feature information across different categories. To better retain distinguishable class characteristics, we adopt learnable temperature scaling alongside individual projection modules tailored for each modality. This design effectively aligns heterogeneous feature spaces. Meanwhile, our framework constrains the semantic consistency of feature embeddings derived from gene expression patterns, subcellular attributes and topological graph structures. Fourth, we design an adaptive fusion module embedded with explicit uncertainty quantification. By evaluating the reliability of different input modalities in real time, this gate mechanism dynamically adjusts the contribution weight of each modal feature during feature aggregation.

## 2. Methods

### 2.1. Problem Formulation

Consider a biological system consisting of *n* proteins, which are formally defined as the set $P=\{p_{1},p_{2},\ldots ,p_{n}\}$. Every protein $p_{i}$ is characterized by three independent feature streams corresponding to different data modalities, each responsible for encoding unique biological attributes of the target protein. Specifically, the gene expression signature of protein $p_{i}$ is formulated as a $d_{g}$-dimensional vector $g_{i}\in R^{d_{g}}$, which quantitatively describes the transcriptional abundance of the coding gene under diverse experimental settings. Subcellular localization feature $s_{i}\in R^{d_{s}}$ is a $d_{s}$-dimensional vector representing the spatial distribution of protein $p_{i}$ across cellular compartments. PPI network node feature $x_{i}\in R^{d_{x}}$ is a $d_{x}$-dimensional vector representing the intrinsic attributes of protein $p_{i}$ in the PPI network. The PPI network is represented as a weighted undirected graph G=(P,E,W), where *E* denotes the set of pairwise protein–protein interactions and $W\in R^{n\times n}$ is the adjacency matrix with $W_{ij}$ representing the confidence score of the interaction between protein $p_{i}$ and $p_{j}$.

The goal of essential protein identification is to learn a predictive function $f:\left(g_{i},s_{i},x_{i},G\right)\to \left\{0,1\right\}$. It maps the multi-modal features and PPI graph structure of protein $p_{i}$ to a binary label, where $y_{i}=1$ indicates an essential protein and $y_{i}=0$ indicates a non-essential protein.

### 2.2. Overview of FusionEP

**Figure 1:**
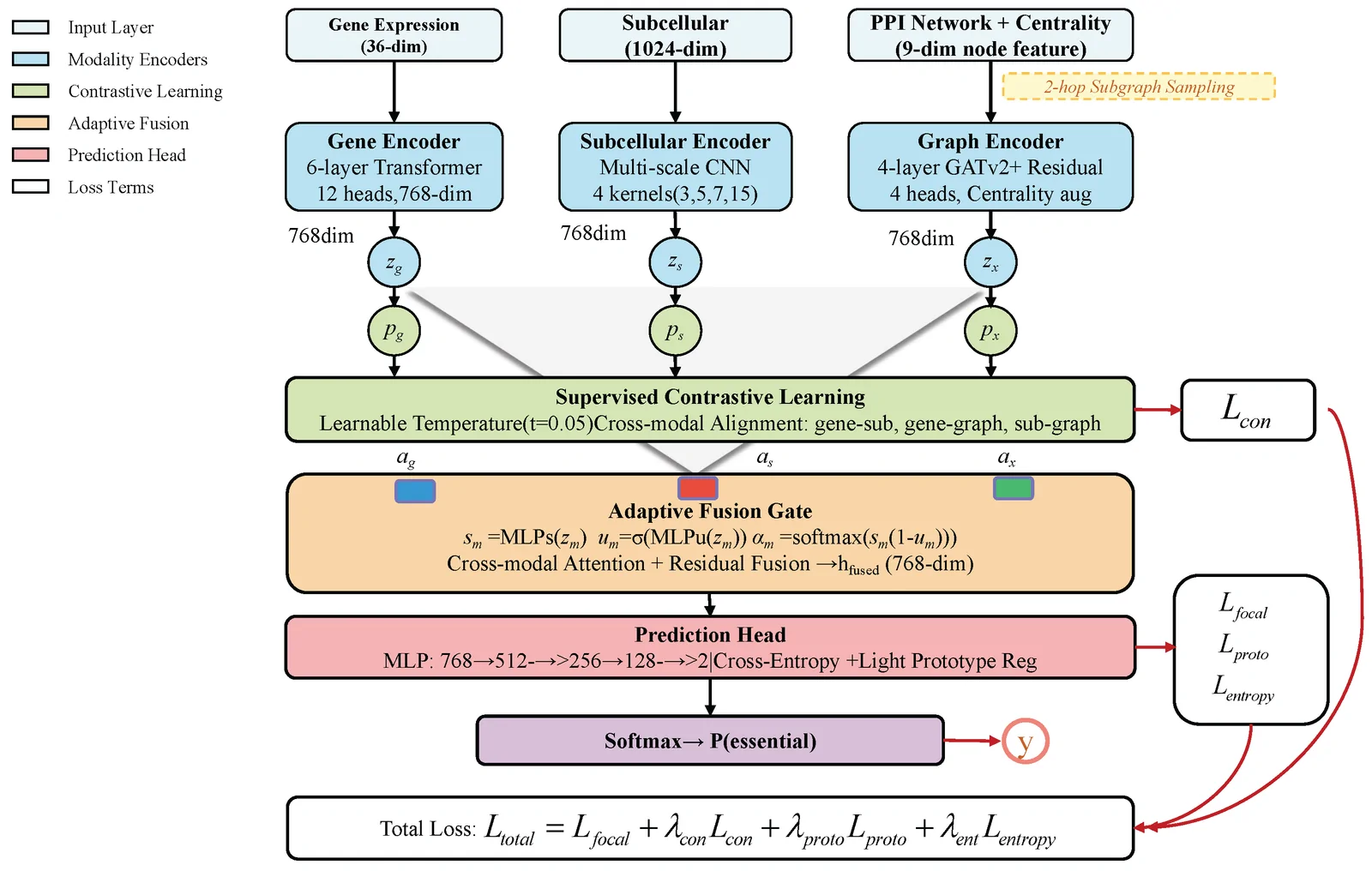
Overview of FusionEP architecture.

**Figure 2:**
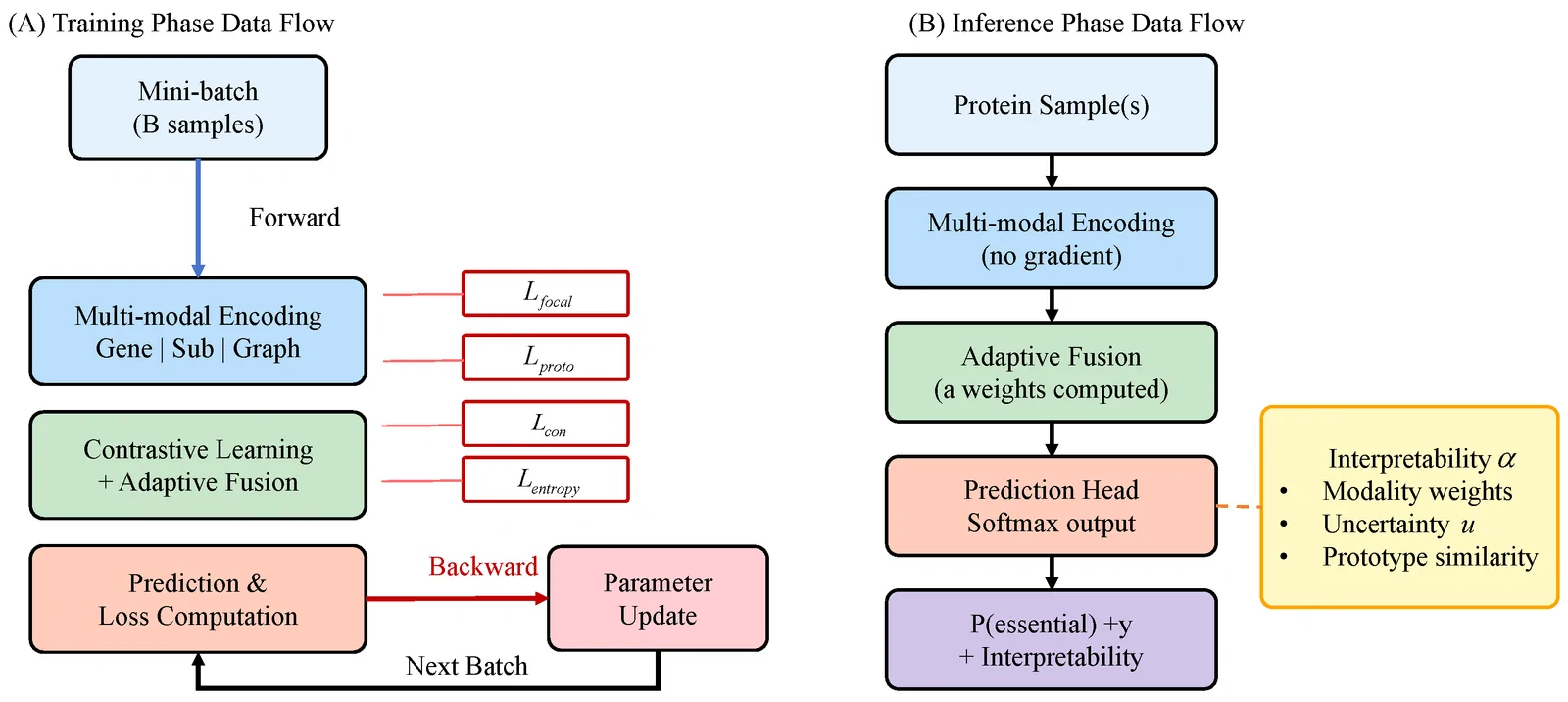
Data flow in FusionEP. (**A**) Training phase. (**B**) Inference phase.

The FusionEP framework consists of five interconnected core components (Figure 1), forming an end-to-end pipeline for multi-modal essential protein prediction. The subgraph sampling component is designed to capture local structural information while ensuring computational efficiency. It samples *k*-hop local subgraphs centered on target proteins and supplements original node features with network centrality indicators, achieving a favorable trade-off between fine-grained local topology modeling and model scalability. In addition, modality-specific encoders adopt dedicated deep learning architectures customized for distinct biological data types, converting raw heterogeneous features from each modality into high-dimensional, discriminative feature embeddings. The enhanced contrastive learning module further bridges the semantic gap across different feature modalities. Equipped with learnable temperature parameters, supervised contrastive learning is applied to drive consistent semantic distribution among multi-modal protein representations and realize effective cross-modal alignment. To fully exploit the complementary advantages of diverse biological features, we develop an adaptive fusion gate with uncertainty perception capability. This module first quantifies the reliability of each modal feature explicitly, then dynamically assigns adaptive weights to different modalities during feature integration, enabling robust and accurate fusion of cross-modal representations. The overall data processing pipeline for model training and inference is visualized in Figure 2. In the training phase, the model takes mini-batch protein samples as input and executes a series of sequential operations including multi-modal feature encoding, cross-modal contrastive constraint, adaptive feature fusion, and final prediction. The entire framework is optimized under a composite loss function composed of four complementary components, i.e., focal loss, contrastive loss, prototype loss, and entropy loss, where gradient backpropagation is implemented to update model parameters jointly. In the inference stage, the optimized model can output both binary classification results and corresponding probability scores to determine the essentiality of target proteins.

### 2.3. Subgraph Sampling with Centrality Augmentation

#### 2.3.1. *k*-Hop Subgraph Extraction

To address scalability limitations of full-batch GNN training, we implement *k*-hop subgraph sampling. For a target protein set $V_{target}$, we iteratively expand the neighborhood by

(1) $$N^{\left(0\right)}=V_{target},\;N^{\left(k\right)}=N^{\left(k-1\right)}\cup \underset{v\in N^{\left(k-1\right)}}{⋃}N^{\left(v\right)},$$

where $N^{\left(v\right)}$ denotes the immediate neighbors of node *v*. We typically set k=2 to balance local structure capture and computational efficiency.

#### 2.3.2. Centrality Feature Computation

We augment raw node features with four network centrality measures computed using NetworkX [18] as

(2) $$\begin{array}{cc} c_{deg}\left(v\right) & \;=\frac{\deg(v)}{n-1}, \end{array}$$

(3) $$\begin{array}{cc} c_{bet}\left(v\right) & \;=\sum_{s\neq v\neq t}\frac{\sigma_{st}\left(v\right)}{\sigma_{st}}, \end{array}$$

(4) $$\begin{array}{cc} c_{clo}\left(v\right) & \;=\frac{n-1}{\sum_{u\neq v}d\left(v,u\right)}, \end{array}$$

(5) $$\begin{array}{cc} c_{eig}\left(v\right) & \;=\frac{1}{\lambda }\sum_{u\in N(v)}c_{eig}\left(u\right), \end{array}$$

where $\sigma_{st}$ is the total number of shortest paths from *s* to *t*, $\sigma_{st}\left(v\right)$ is the number passing through *v*, d(v,u) is the shortest path distance, and λ is the largest eigenvalue of the adjacency matrix.

The final node feature is the concatenation by

(6) $$x_{i}^{aug}=\left[x_{i};c_{deg};c_{bet};c_{clo};c_{eig}\right]\in R^{d_{x}+4}.$$

### 2.4. Modality-Specific Encoders

**Figure 3:**
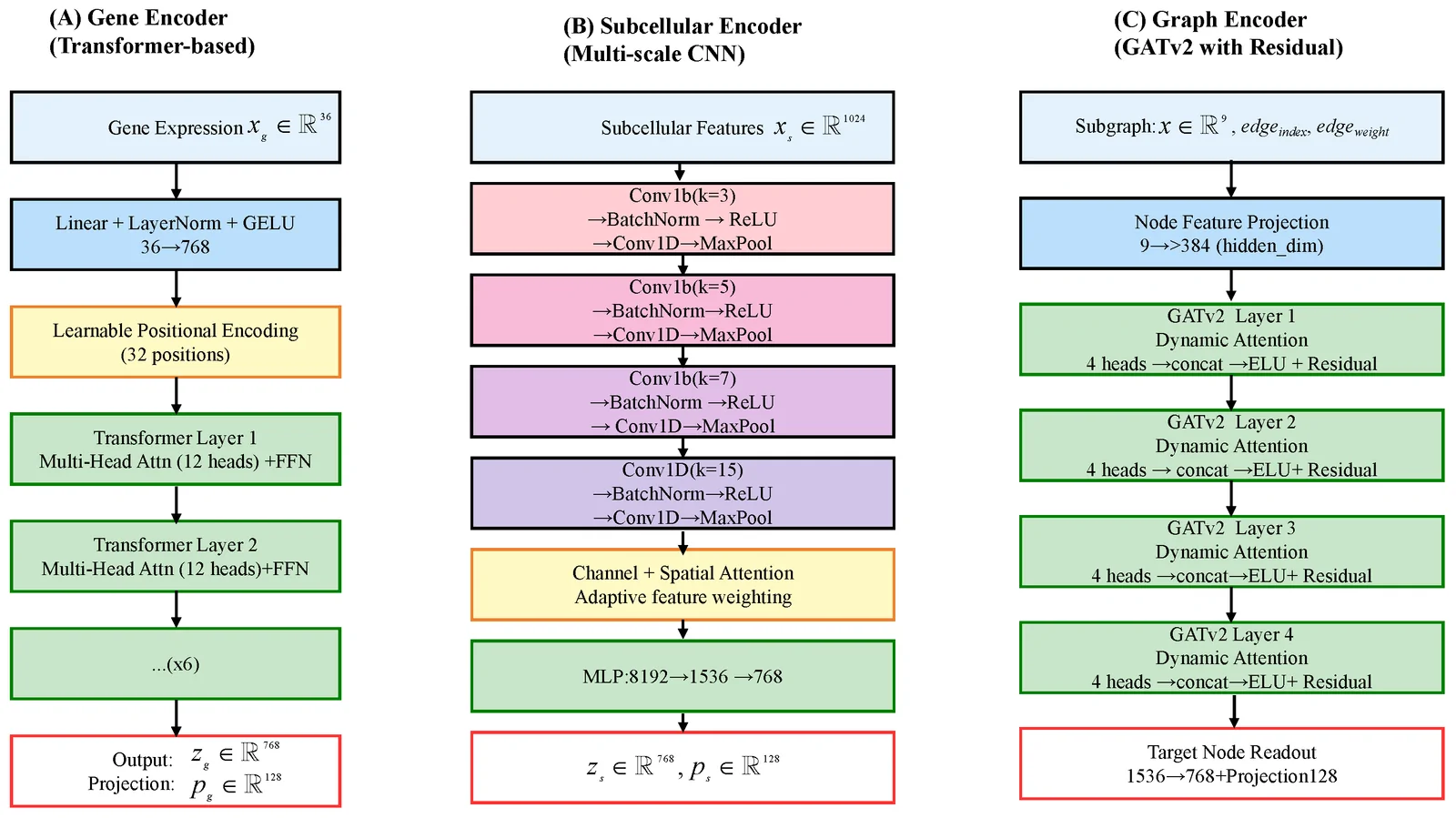
Detailed architecture of modality-specific encoders. (**A**) Gene encoder: 6-layer Transformer with 12 attention heads and learnable positional encoding for gene-expression feature extraction. (**B**) Subcellular encoder: multi-scale one-dimensional CNN with four kernel sizes (3, 5, 7, 15), combined with channel-spatial attention for subcellular-localization feature learning. (**C**) Graph encoder: 4-layer GATv2 with dynamic attention, residual connections, and target-node readout, which takes centrality-augmented subgraph node features as input. Each encoder outputs a 768-dimensional representation and a 128-dimensional projection for contrastive learning.

We design three specialized deep encoders to transform raw features from gene expression, subcellular localization, and PPI graph modalities into high-dimensional discriminative representations (Figure 3). Each encoder is tailored to the unique structural characteristics of its corresponding data type to ensure optimal feature extraction.

#### 2.4.1. Gene Expression Encoder

Gene expression data $g\in R^{d_{g}}$ is processed through a deep Transformer encoder as

(7) $$\begin{array}{cc} h_{g}^{\left(0\right)} & \;=\mathrm{Linear}\left(g\right)+E_{pos}, \end{array}$$

(8) $$\begin{array}{cc} h_{g}^{\left(l\right)} & \;=\mathrm{TransformerLayer}(h_{g}^{\left(l-1\right)}),\;l=1,\ldots ,L, \end{array}$$

(9) $$\begin{array}{cc} z_{g} & \;=\mathrm{MeanPool}(h_{g}^{\left(L\right)}), \end{array}$$

(10) $$\begin{array}{cc} p_{g} & \;=\mathrm{Normalize}(\mathrm{MLP}\left(z_{g}\right)), \end{array}$$

where $E_{pos}$ represents learnable positional encodings, L=6 layers, and $p_{g}\in R^{128}$ is the contrastive projection.

#### 2.4.2. Subcellular Localization Encoder

Subcellular features $s\in R^{d_{s}}$ are processed by a multi-scale CNN with attention mechanisms by

(11) $$\begin{array}{cc} h_{s}^{\left(k\right)} & \;={\mathrm{Conv}1D}_{k}\left(s\right),\;k\in \left\{3,5,7,15\right\}, \end{array}$$

(12) $$\begin{array}{cc} h_{s}^{cat} & \;=\mathrm{Concat}(\left[h_{s}^{\left(k\right)}\right]), \end{array}$$

(13) $$\begin{array}{cc} h_{s}^{attn} & \;=h_{s}^{cat}⊙\sigma \left(\mathrm{ChannelAttn}\left(h_{s}^{cat}\right)\right)⊙\sigma \left(\mathrm{SpatialAttn}\left(h_{s}^{cat}\right)\right), \end{array}$$

(14) $$\begin{array}{cc} z_{s} & \;=\mathrm{MLP}(\mathrm{Flatten}\left(h_{s}^{attn}\right)), \end{array}$$

(15) $$\begin{array}{cc} p_{s} & \;=\mathrm{Normalize}(\mathrm{MLP}\left(z_{s}\right)). \end{array}$$

#### 2.4.3. Graph Encoder with GATv2

The graph encoder processes subgraphs using GATv2 [19] with residual connections as

(16) $$\begin{array}{cc} h_{v}^{\left(0\right)} & \;=\mathrm{Linear}(x_{v}^{aug}), \end{array}$$

(17) $$\begin{array}{cc} h_{v}^{\left(l\right)} & \;=\mathrm{ELU}\left({\mathrm{GATv}\;2}^{\left(l\right)}\left(h^{\left(l-1\right)},E\right)\right)+h_{v}^{\left(l-1\right)}, \end{array}$$

(18) $$\begin{array}{cc} z_{v}^{graph} & \;=\mathrm{Readout}(h_{v}^{\left(L\right)}), \end{array}$$

(19) $$\begin{array}{cc} p_{v}^{graph} & \;=\mathrm{Normalize}(\mathrm{MLP}\left(z_{v}^{graph}\right)), \end{array}$$

where GATv2 employs dynamic attention $\alpha_{ij}={\mathrm{Softmax}}_{j}\left(a^{⊤}\mathrm{LeakyReLU}\left(W\left[h_{i}∥h_{j}\right]\right)\right)$.

### 2.5. Enhanced Supervised Contrastive Learning

We propose an enhanced supervised contrastive learning module with learnable temperature scaling and modality-specific alignment projections.

#### 2.5.1. Cross-Modal Alignment

For each modality pair $\left(m_{i},m_{j}\right)\in \left\{\left(\mathrm{gene},\mathrm{sub}\right),\left(\mathrm{gene},\mathrm{graph}\right),\left(\mathrm{sub},\mathrm{graph}\right)\right\}$, we compute aligned projections

(20) $$q_{i}^{\left(ij\right)}={\mathrm{MLP}}_{ij}\left(p_{i}\right),\;q_{j}^{\left(ij\right)}={\mathrm{MLP}}_{ij}\left(p_{j}\right).$$

#### 2.5.2. Supervised Contrastive Loss

The similarity matrix is computed as

(21) $$S_{ij}=\frac{q_{i}^{\left(ij\right)}\cdot {\left(q_{j}^{\left(ij\right)}\right)}^{⊤}}{\tau },\;\tau =\frac{1}{\exp(\beta )},$$

where β is a learnable parameter controlling temperature.

For samples with labels y, we define positive and negative masks as

(22) $$\begin{array}{cc} M_{pos} & \;=⊮\left(y_{i}=y_{j}\right)⊙\left(1-I\right), \end{array}$$

(23) $$\begin{array}{cc} M_{neg} & \;=⊮(y_{i}\neq y_{j}). \end{array}$$

The contrastive loss for a pair is

(24) $$L_{con}^{\left(ij\right)}=-\frac{1}{N}\sum_{i}\log\frac{\sum_{j}\exp\left(S_{ij}\right)⊙M_{pos}}{\sum_{j}\exp\left(S_{ij}\right)⊙\left(M_{pos}+M_{neg}\right)}.$$

Total contrastive loss $L_{con}=\frac{1}{3}\sum_{pairs}L_{con}^{\left(ij\right)}$.

### 2.6. Adaptive Uncertainty-Aware Fusion

The adaptive fusion gate computes modality-importance weights with explicit uncertainty estimation by

(25) $$\begin{array}{cc} s_{m} & \;={\mathrm{MLP}}_{s}\left(z_{m}\right),\;m\in \left\{g,s,x\right\}, \end{array}$$

(26) $$\begin{array}{cc} u_{m} & \;=\sigma ({\mathrm{MLP}}_{u}\left(z_{m}\right)), \end{array}$$

(27) $$\begin{array}{cc} {\tilde{s}}_{m} & \;=s_{m}\cdot \left(1-u_{m}\right), \end{array}$$

(28) $$\begin{array}{cc} \alpha_{m} & \;=\frac{\exp({\tilde{s}}_{m})}{\sum_{k}\exp\left({\tilde{s}}_{k}\right)}, \end{array}$$

where $s_{m}$ denotes the raw importance score for modality *m*, and $u_{m}\in \left[0,1\right]$ is the corresponding modality-level uncertainty estimate. Higher uncertainty suppresses the effective importance score ${\tilde{s}}_{m}$ prior to softmax normalization to produce the final modality weight $\alpha_{m}$.

Cross-modal multi-head attention captures interactions across modality-specific representations. It treats $\left[z_{g},z_{s},z_{x}\right]$ as an input sequence of length three and outputs three context-aware vectors $h_{g}^{\mathrm{attn}}$, $h_{s}^{\mathrm{attn}}$, and $h_{x}^{\mathrm{attn}}$. Each output vector $h_{m}^{\mathrm{attn}}$ corresponds to the *m*-th position in the output sequence and aggregates information from all three input modalities:

(29) $$h_{m}^{\mathrm{attn}}=\sum_{k\in \{g,s,x\}}\mathrm{Attn}\left(m,k\right)\cdot v_{k},$$

where Attn(m,k) is the learned attention weight between query from modality *m* and key from modality *k*, and $v_{k}$ denotes the value vector linearly projected from input representation $z_{k}$. Consequently, each $h_{m}^{\mathrm{attn}}$ integrates cross-modal information from gene, subcellular-localization, and PPI-graph representations.

Each modality representation is individually transformed and combined with its corresponding attention context to obtain modality-aware representations:

(30) $${\tilde{h}}_{m}=\mathrm{MLP}\left(z_{m}\right)+h_{m}^{\mathrm{attn}}.$$

The final fused representation is the weighted sum over modality-aware outputs using uncertainty-calibrated scalar weights:

(31) $$h_{\mathrm{out}}=\sum_{m\in \{g,s,x\}}\alpha_{m}\cdot {\tilde{h}}_{m}.$$

In this formulation, weights $\alpha_{m}$ modulate each modality’s own transformed representation, avoiding the mathematical flaw of weighting a shared identical pre-fused vector. A higher uncertainty estimate reduces the contribution of the corresponding modality to the final fused representation.

### 2.7. Prediction Head with Focal Loss

The prediction head employs focal loss to address class imbalance by

(32) $$L_{focal}=-\sum_{i}\alpha_{y_{i}}{\left(1-p_{i,y_{i}}\right)}^{\gamma }\log\left(p_{i,y_{i}}\right),$$

where α=[0.25,0.75] for class weights, γ=2 is the focusing parameter, and $p_{i,y_{i}}$ is the predicted probability for the true class.

Additionally, we maintain learnable class prototypes $c_{k}$ and compute prototype contrastive loss

(33) $$L_{proto}=-\log\frac{\exp(\mathrm{sim}\left(h_{out},c_{y_{i}}\right))}{\sum_{k}\exp\left(\mathrm{sim}\left(h_{out},c_{k}\right)\right)}.$$

Furthermore, we adopt entropy regularization $L_{entropy}$ to encourage the model to produce sharp and confident predictions, reducing predictive uncertainty and enhancing representation discriminability. Given the predicted probability distribution $p_{i}=\left[p_{i,1},p_{i,2}\right]$ of protein *i* over two categories, the entropy loss is formulated as

(34) $$L_{entropy}=-\frac{1}{N}\sum_{i=1}^{N}\sum_{k=1}^{2}p_{i,k}\log\left(p_{i,k}\right).$$

Total training loss

(35) $$L_{total}=L_{focal}+\lambda_{con}L_{con}+\lambda_{proto}L_{proto}+\lambda_{ent}L_{entropy}.$$

## 3. Experimental Setup

### 3.1. Dataset

Protein-protein interaction data were retrieved from the BioGRID database (https://thebiogrid.org/ (accessed on 12 Septempber 2023)) [20] after removing redundant records. The final yeast protein–protein interaction dataset contained 5616 entries. Essential proteins in the interaction network were identified by integrating data from SSGD [21], MIPS [22], DEG [23], and SGDP [24], yielding 1199 essential proteins. Corresponding gene expression profiles were obtained from the Gene Expression Omnibus (GEO, https://www.ncbi.nlm.nih.gov/geo/ (accessed on 12 Septempber 2023)) [25], while subcellular localization annotations were collected from the COMPARTMENTS database (https://compartments.jensenlab.org/ (accessed on 12 Septempber 2023)) [26]. The raw PPI data from BioGRID were pre-processed to remove redundant entries, self-loop interactions and low-confidence edges with weak experimental evidence. Interaction confidence scores were mapped to edge weights of the undirected graph (G = (P,E,W)). Edge weights are consumed within the GATv2 graph encoder during dynamic-attention computation rather than used as node-level features. Unique protein and edge counts after filtering are 5616 unique proteins and 21,478 filtered non-redundant interactions. Protein identifiers from different annotation databases were mapped via UniProt ID conversion. For missing entries in gene-expression or subcellular features, zero-value imputation was applied.

### 3.2. Implementation Details

The model is implemented in PyTorch 1.12+ with PyTorch Geometric. Key hyperparameters are listed in Table 1.

We employ stratified train/validation/test splits (70%/15%/15%) and report metrics averaged over five independent runs with different random seeds.

### 3.3. Evaluation Metrics

We evaluate the performance of the proposed model using AUC-ROC (area under the receiver operating characteristic curve), AUC-PR (area under the precision–recall curve ), F1 score (harmonic mean of precision and recall), MCC (Matthews correlation coefficient).

### 3.4. Baseline Methods

We compare against the DC (Degree Centrality) [3], BC (Betweenness Centrality) [5], CC (Closeness Centrality) [6], EC (Eigenvector Centrality) [7], AttentonEP [15], DeepEP [12], and ECDEP [14] of multi-information fusion.

## 4. Results

### 4.1. Overall Performance

Table 2 summarizes the complete quantitative metrics for various methods. FusionEP achieves state-of-the-art performance across all evaluation metrics. AUC-ROC is 0.9523 ± 0.003. AUC-PR is 0.8234 ± 0.005, and F1 score is 0.8567 ± 0.004. MCC is 0.8123 ± 0.004. Classical topological centrality methods (DC/BC/CC/EC) achieve the lowest performance (AUC-ROC < 0.75). This means that a single topological feature is insufficient for accurate essential protein prediction. FusionEP not only achieved the best AUC-ROC and AUC-PR results but also maintained good performance for F1 score and MCC. This indicates that the model has excellent class imbalance handling capabilities and strong generalization ability for unknown protein samples.

### 4.2. Ablation Studies

To quantitatively validate the contribution and necessity of each core module embedded in the proposed FusionEP model, we carry out a series of systematic ablation experiments. We implement module elimination and replacement experiments to analyze the performance variations brought by each key component. To guarantee rigorous and fair comparative analysis, all ablation tests were conducted under identical experimental configurations and hyperparameter settings. The detailed quantitative results presented in Table 3 clearly validate the indispensable function of each designed module in boosting the overall prediction performance. First, we substitute the customized sampling strategy with random subgraph sampling for comparison. The modified model yields a noticeable AUC-ROC drop of 4.12%, declining from the original 0.9523 to 0.9135. This outcome proves that the integration of network centrality attributes and local subgraph structural information enables the model to precisely capture the global topological significance of target proteins in PPI networks. Second, we remove the cross-modal contrastive learning module from the overall framework. This modification leads to a sharp performance degradation, with the AUC-ROC value falling by 5.47% to 0.8976. This significant performance gap verifies that cross-modal semantic alignment serves as a core foundation for fusing heterogeneous multi-omics biological data. By unifying feature distribution from different modalities into a shared semantic space, this module effectively eliminates feature heterogeneity discrepancies. Third, we replace the adaptive uncertainty-aware fusion mechanism with a static equal-weight fusion strategy, which assigns identical weights to all modal features without considering data quality differences. The resulting AUC-ROC decreases by 3.85% to 0.9158. This comparison strongly supports the superiority of our dynamic weight adjustment design. The adaptive mechanism can rationally allocate modal weights according to the real-time reliability of input data, thereby greatly enhancing the model’s robustness against biological noise and incomplete data samples. Furthermore, we discard the prototype regularization strategy and prototype loss constraint in the model training process. The experimental results show that the AUC-ROC drops by 2.31% to 0.9305, accompanied by a 1.89% reduction in F1 score. It is evident that prototype regularization assists the model in learning more distinguishable feature patterns, strengthening the classification capability between essential and non-essential proteins. Finally, we disable all modality-specific data augmentation operations in the training pipeline. The model achieves an AUC-ROC of 0.9327, representing a 2.10% performance decline. This result indicates that the designed data augmentation schemes are effective in improving the model’s generalization capacity, allowing the model to adapt better to complex and noisy real-world biological data.

## 5. Discussion

Extensive experimental results fully demonstrate that FusionEP outperforms existing state-of-the-art baseline methods across all evaluation metrics. The model exhibits excellent stability and robustness against biological noise data. Its outstanding performance is attributed to the synergistic effect of its five core components. Each component specifically addresses various core shortcomings of traditional protein prediction methods.

A centrality-enhanced k-hop subgraph sampling strategy effectively addresses the scalability challenge of full-batch GNN training on large-scale PPI networks. This method extracts local subgraphs and combines them with global centrality features to enhance features. The model thus balances computational efficiency and topological information learning. Existing GNN methods generally suffer from the difficulty of balancing performance and efficiency. Performing full-network analysis with these methods sacrifices scalability. Using simple subgraph sampling results in the loss of global topological information. Ablation experiments demonstrate that this module can improve AUC-ROC by 4.12%. This result indicates that this strategy can accurately capture globally important protein features while ensuring the feasibility of training the model on large-scale proteomics data.

Modality-specific encoders can fully exploit the unique structural features of various biological data. Transformer-based gene expression encoders can effectively capture contextual relationships within experimental conditions. Multi-scale CNNs with attention mechanisms can extract fine-grained spatial features from subcellular localization data. GATv2 graph encoders with residual connections can handle heterogeneous interaction weights in PPI networks. This model employs a modality-adaptive, customized encoding design. Existing multimodal methods often use general encoders to process all data modalities. This makes it difficult to accurately capture the unique biological characteristics specific to different modalities.

The enhanced supervised contrastive learning module effectively addresses the heterogeneity challenge of integrating multi-omics data. This module achieves cross-modal semantic alignment. Existing methods only splice or linearly fuse multimodal features. FusionEP maps gene expression, subcellular localization, and PPI network features to a unified semantic space. It mainly employs learnable alignment projection and temperature scaling strategies. Ablation experiments show that removing this module reduces the model’s AUC-ROC by 5.47%. This demonstrates that cross-modal alignment can efficiently fuse heterogeneous biological data. It can fully exploit the complementary information between different modalities.

The prototype regularization prediction head combined with focal loss. It effectively alleviates the class imbalance problem in essential protein datasets. Prototype regularization enhances the model’s class discrimination learning ability. Focal loss concentrates training on hard-to-learn essential proteins.

### 5.1. Differences from Classical Topological Methods

Traditional topological centrality metrics (DC, BC, CC, EC) make predictions exclusively using manually defined topological statistics derived from PPI networks. A well-known limitation is that many essential proteins do not occupy high-centrality positions in the interaction graph, while a large number of hub proteins are non-essential. Topology-only strategies struggle to separate these two groups reliably. FusionEP incorporates multiple complementary biological signals: gene expression measurements and subcellular positional information. Its modality-specific encoders learn nonlinear discriminative representations from each data source. Supervised contrastive learning projects these heterogeneous features into a common semantic space, and the uncertainty-aware fusion gate assigns sample-specific weights to balance the input modalities. This design accounts for why FusionEP achieves better predictive performance and can yield predictions different from those generated by topological baseline methods.

### 5.2. Interpretation of Adaptive Uncertainty-Aware Fusion

**Figure 4:**
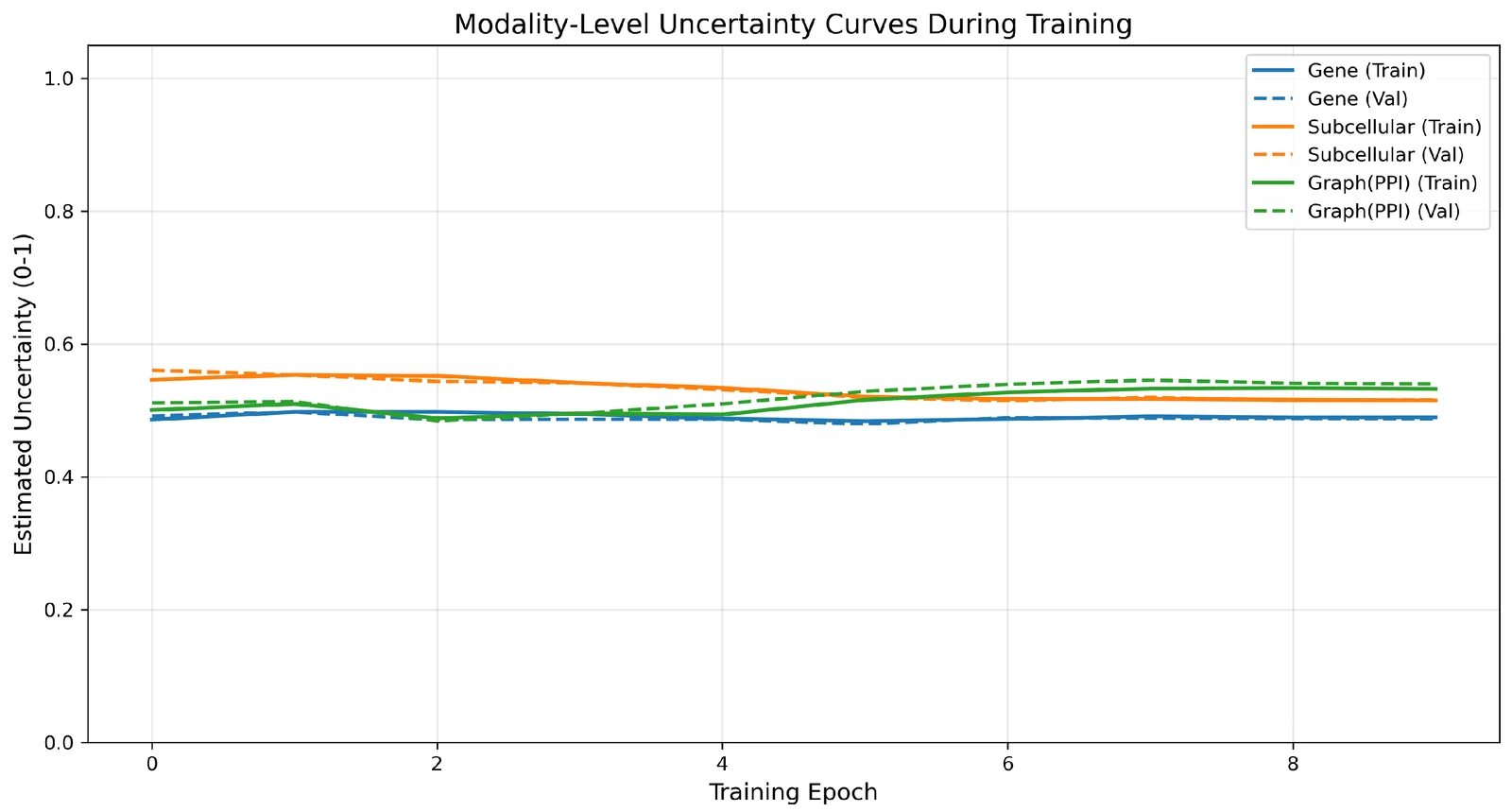
Modality-level uncertainty dynamics across training epochs. Estimated uncertainty values (range [0, 1]) for gene-expression, subcellular-localization, and PPI-graph modalities on the validation set. Higher uncertainty indicates lower reliability of the corresponding modality, which will be suppressed by the adaptive fusion gate during feature aggregation.

We further examine FusionEP’s adaptive fusion behavior by visualizing how uncertainty estimates for each modality evolve across training epochs (Figure 4). Separate uncertainty estimators are assigned to the three input sources: gene expression, subcellular localization and the PPI graph. At the start of training, the subcellular localization modality carries the largest uncertainty value, which gradually falls as training proceeds. For the PPI graph modality, uncertainty first decreases before showing a sustained upward trend. In comparison, gene expression maintains consistently low uncertainty over the whole training process. These observed trends demonstrate that FusionEP adjusts cross-modal fusion weights dynamically according to each modality’s uncertainty. Features with higher uncertainty contribute less to the final prediction. In stark contrast, classical topological centrality metrics (DC, BC, CC, EC) depend on static PPI network topology with fixed hand-engineered rules. They cannot account for noise or varying reliability across multi-omics datasets. This core distinction enables FusionEP to capture complementary biological cues and recover essential proteins missed by topology-only algorithms.

### 5.3. Biological Interpretation

The learned modality weights endow the model with biological interpretability. For most essential proteins, both gene expression and graph structure modalities receive high weights. For a subset of essential proteins with low centrality in PPI networks, subcellular localization and gene expression signals dominate predictions. Model outputs such as modality weights and uncertainty estimates help rank candidate proteins for wet-lab verification and therapeutic target discovery.

### 5.4. Limitations

This study has clear limitations. First, all validations are conducted only on yeast, thus its cross-species generalization needs further testing. Second, FusionEP requires complete gene expression, subcellular localization and PPI data. Its prediction performance may drop substantially if a modality contains extensive missing values. Third, the adopted two-hop subgraph sampling strategy may lose partial long-range global topological signals. Fourth, the multi-encoder and contrastive-learning modules bring non-trivial computational overhead compared with simple topological metrics. Future improvements will focus on modality-missing robust training, more efficient graph-sampling strategies, and cross-species transfer-learning frameworks.

## 6. Conclusions

In this work, we proposed FusionEP to identify essential proteins. It’s an adaptive cross-modal deep fusion network. It integrates gene expression profiles, subcellular localization features, and PPI network topology. A *k*-hop subgraph sampling strategy is used to capture global topological features. A dedicated encoder for each biological modality is proposed. Cross-modal contrastive learning is adopted to preserve class distinction information. The adaptive fusion gate with uncertainty estimation is set. This approach collaboratively addresses the shortcomings of existing methods. The study conducted experimental evaluations on the BioGRID dataset. FusionEP achieved state-of-the-art performance across all metrics. Its AUC-ROC was 0.9523 ± 0.003, AUC-PR was 0.8234 ± 0.005, F1 score was 0.8567 ± 0.004, and MCC was 0.8123 ± 0.004. Ablation studies validated the effectiveness of each core component. FusionEP provides an efficient end-to-end solution for identifying essential proteins. It has potential applications in systems biology, drug discovery, and precision medicine.

## Figures and Tables

**Table 1:** Hyperparameter configuration.

Parameter	Value
Embedding dimension	768
Gene encoder depth	6 layers
Gene attention heads	12
Subcellular scales	4 (kernels 3, 5, 7, 15)
Graph layers	4 GATv2 layers
Graph attention heads	4
Subgraph hops (*k*)	2
Optimizer	AdamW
Weight-decay	$10^{-4}$
Learning rate (gene)	$5\times 10^{-5}$
Learning rate (sub/graph)	$1\times 10^{-4}$
Learning rate (fusion/predictor)	2–$3\times 10^{-4}$
Batch size	64
Epochs	100
Patience (early stopping)	30
Temperature τ	0.05 (learnable)
Contrastive weight $\lambda_{con}$	1.0
$\lambda_{proto}$	0.5
$\lambda_{ent}$	0.1
Dropout rate	0.2–0.35

**Table 2:** Performance comparison on BioGRID dataset (mean ± std over 5 runs).

Method	AUC-ROC	AUC-PR	F1	MCC
DC	0.7234 ± 0.012	0.3456 ± 0.018	0.4123 ± 0.015	0.2845 ± 0.014
BC	0.7456 ± 0.011	0.3678 ± 0.016	0.4356 ± 0.013	0.3123 ± 0.012
CC	0.7123 ± 0.013	0.3345 ± 0.019	0.3987 ± 0.016	0.2678 ± 0.015
EC	0.7345 ± 0.012	0.3567 ± 0.017	0.4234 ± 0.014	0.2956 ± 0.013
AttentionEP	0.9407 ± 0.003	0.8779 ± 0.009	0.8071 ± 0.010	0.7550 ± 0.016
DeepEp *	0.82	0.61	0.55	0.44
ECDEP *	0.92	0.86	0.80	0.77
FusionEP (Ours)	0.9523 ± 0.003	0.8234 ± 0.005	0.8567 ± 0.004	0.8123 ± 0.004

* The results with asterisk are quoted from references [12,14].

**Table 3:** Ablation study of FusionEP core components (AUC-ROC, mean ± std).

Model Variant	AUC-ROC
FusionEP (Full Model)	0.9523 ± 0.003
− Centrality-augmented subgraph sampling	0.9135 ± 0.004
− Cross-modal contrastive learning	0.8976 ± 0.005
− Adaptive uncertainty-aware fusion (static weighting)	0.9158 ± 0.004
− Prototype regularization	0.9305 ± 0.003
− Data augmentation	0.9327 ± 0.003

## Data Availability

The original data presented in the study are openly available in https://github.com/biospacecy/FusionEP, accessed on 12 Septempber 2023.

## Abbreviations

The following abbreviations are used in this manuscript:

AUC-ROC	Area Under the Receiver Operating Characteristic Curve
AUC-PR	Area Under the Precision-Recall Curve
BC	Betweenness Centrality
CC	Closeness Centrality
CNN	Convolutional Neural Network
CRISPR	Clustered Regularly Interspaced Short Palindromic Repeats
DC	Degree Centrality
DEG	Database of Essential Genes
EC	Eigenvector Centrality
GAT	Graph Attention Network
GATv2	Graph Attention Network version 2
GCN	Graph Convolutional Network
GEO	Gene Expression Omnibus
GNN	Graph Neural Network
MCC	Matthews Correlation Coefficient
MIPS	Munich Information Center for Protein Sequences
PPI	Protein-Protein Interaction
RNAi	RNA Interference
SGDP	Saccharomyces Genome Deletion Project
SGD	Saccharomyces Genome Database
